# Role of post-translational modifications in modulating histone reactivity in the extracellular environment

**DOI:** 10.1042/BST20250113

**Published:** 2026-07-30

**Authors:** Helen Hemmling, Clare L. Hawkins

**Affiliations:** Department of Biomedical Sciences, University of Copenhagen, Panum, Blegdamsvej 3B, Copenhagen N DK-2200, Denmark

**Keywords:** extracellular trap, histones, inflammation, neutrophils, post translational modification

## Abstract

Histones are critical for the packaging of nuclear DNA and chromatin assembly, which is facilitated by the high abundance of lysine and arginine residues within these proteins. These residues are the site of post-translational modifications, which regulate cellular processes involving DNA, such as transcription, replication, and repair. Histones are also present in the extracellular environment, following their passive release by dying cells or active release by immune cells as extracellular traps. In the extracellular environment, histones are potent antimicrobial agents and play a role in limiting the spread of infection. However, there is strong evidence that extracellular histones are also involved in propagating disease, owing to their damaging reactions with host cells. Histones are cytotoxic and pro-inflammatory and drive coagulation, which has been associated with organ failure and death in both acute and chronic inflammatory diseases. The present review describes the reactivity of histones in the extracellular environment, with a particular focus on how these reactions are influenced by post-translational modifications relevant to physiological and pathological conditions.

## Introduction

Histones are best known for their fundamental role in maintaining chromatin structure in the cell nucleus by packaging DNA into nucleosomes, as well as their role in gene expression, where they control the accessibility of DNA to transcription factors through different post-translational modifications (PTMs) [1–3]. Each nucleosome consists of DNA (ca. 147 base pairs) wrapped twice around an octomer of core histones comprised of two H2A–H2B dimers and a tetramer of H3–H4, which are assembled into ordered chromatin by the linker histone, H1 [1–3]. Histones are strongly basic proteins, on account of their high abundance of Lys and Arg residues. This is critical to their role in packaging and stabilising the negatively charged DNA. These residues are subject to a wide range of PTMs, including acetylation, methylation, phosphorylation, citrullination, SUMOylation, ubiquination, and ADP-ribosylation, which influence the extent of histone binding to DNA and hence the folding of chromatin [4]. Combinations of these PTMs in specific genomic regions are known as the ‘histone code’, which ultimately activates or represses gene expression [5,6].

Recent studies show that histones are also susceptible to a range of oxidative modifications, which could be important in influencing their reactivity, particularly when outside the cell [7–11]. Thus, the release of histones into the extracellular environment, because of cell damage or activation, drives toxicity and exacerbates inflammation and coagulation [12–14]. Interestingly, these damaging properties of histones are also related to their highly basic nature, which facilitates binding to cell membranes and/or surface receptors [14,15]. As such, it is widely accepted that histones in the extracellular environment act as damage associated molecular patterns (DAMPs) [16,17] and are strongly linked to the pathophysiology of many diseases [14,18,19]. The present review will focus on the extracellular reactivity of histones, including how these reactions are influenced by PTMs relevant to pathological conditions. These reactions could be important to consider to better understand disease mechanisms and the design of new therapeutic strategies to modulate histone-induced damage in a wide range of pathologies.

## Extracellular release of histones from cells

**Figure 1:**
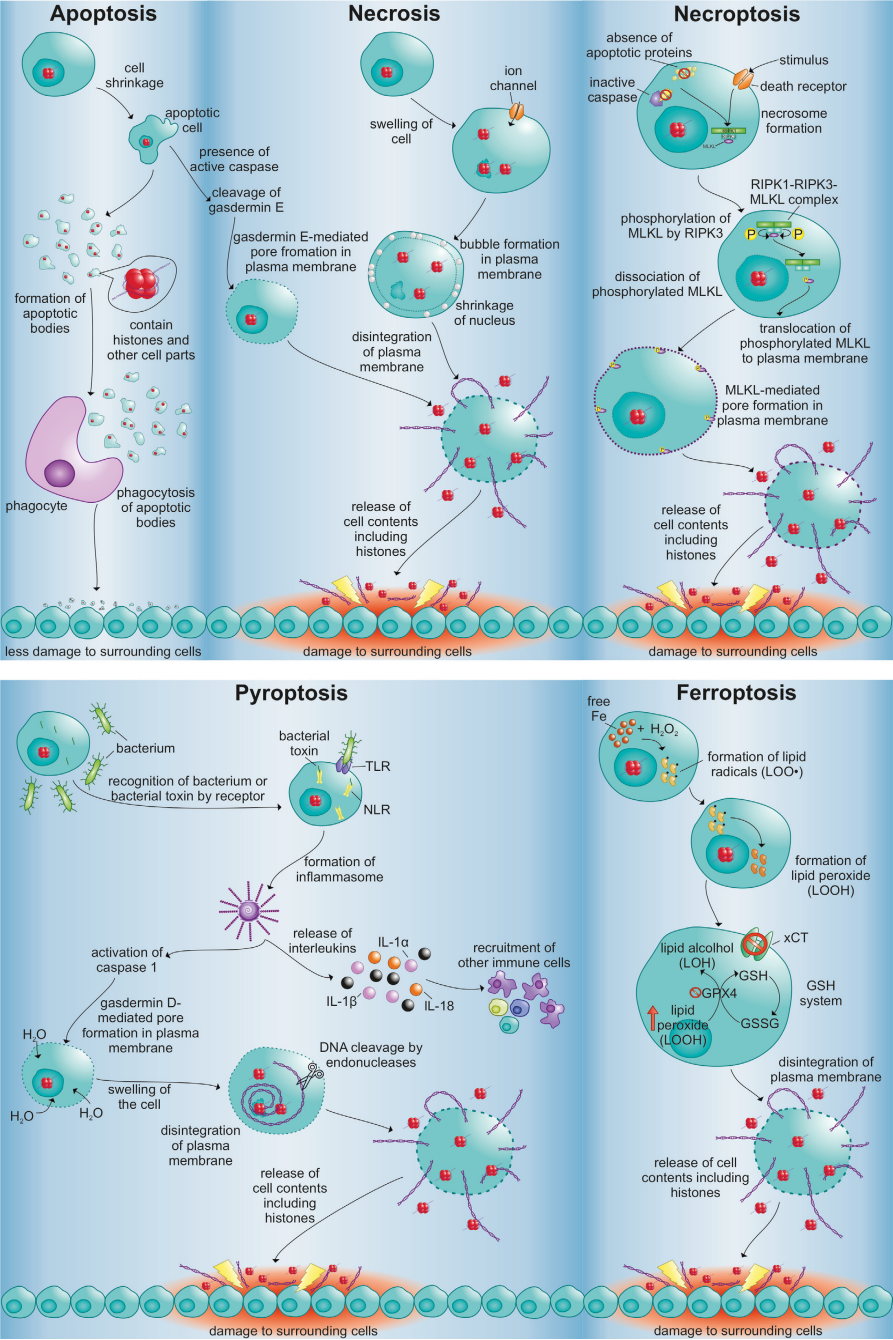
Release of histones via different cell death pathways Histones can be released to the extracellular environment by different cell death pathways, which can have pro-inflammatory and damaging effects on neighbouring cells. The figure was created with CorelDraw® 2019, version 21.2.0.708.

**Figure 2:**
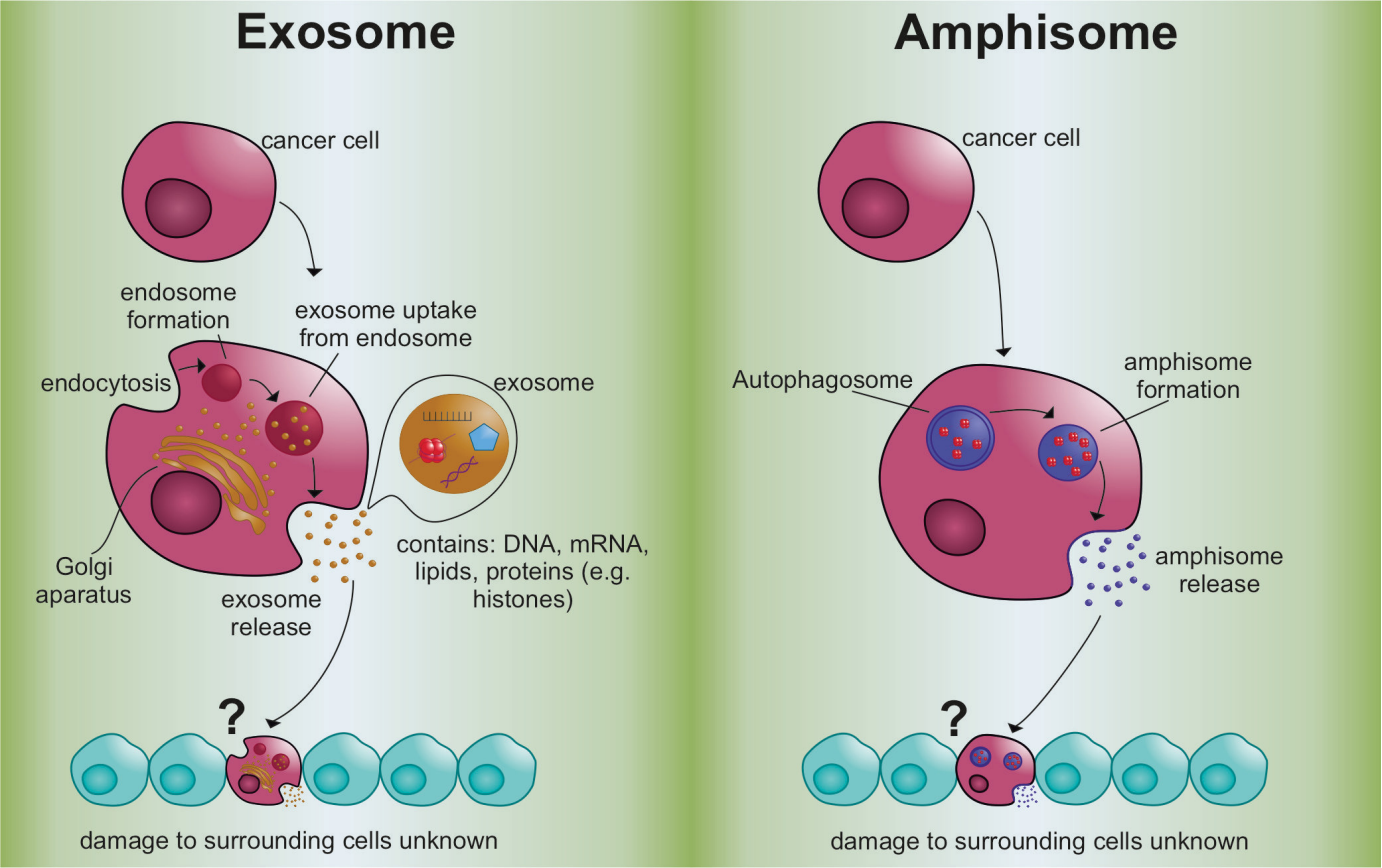
Histone release from cancer cells Cancer cells can release histones via two different pathways: (1) formation of exosomes or (2) formation of amphisomes. (1) Exosomes are small extracellular vesicles that originate from the inward invert of endosomal membranes. They contain DNA, mRNA, lipids, and proteins (e.g. histones) and, once taken up into endosomes, can be released via exocytosis. (2) Histone release due to amphisome formation is possible, as histones are released from the nucleus in multivesicular bodies and fuse with autophagosomes, forming amphisomes. Amphisomes fuse then with the plasma membrane and then release their content including the histones. The figure was created with CorelDraw® 2019, version 21.2.0.708.

**Figure 3:**
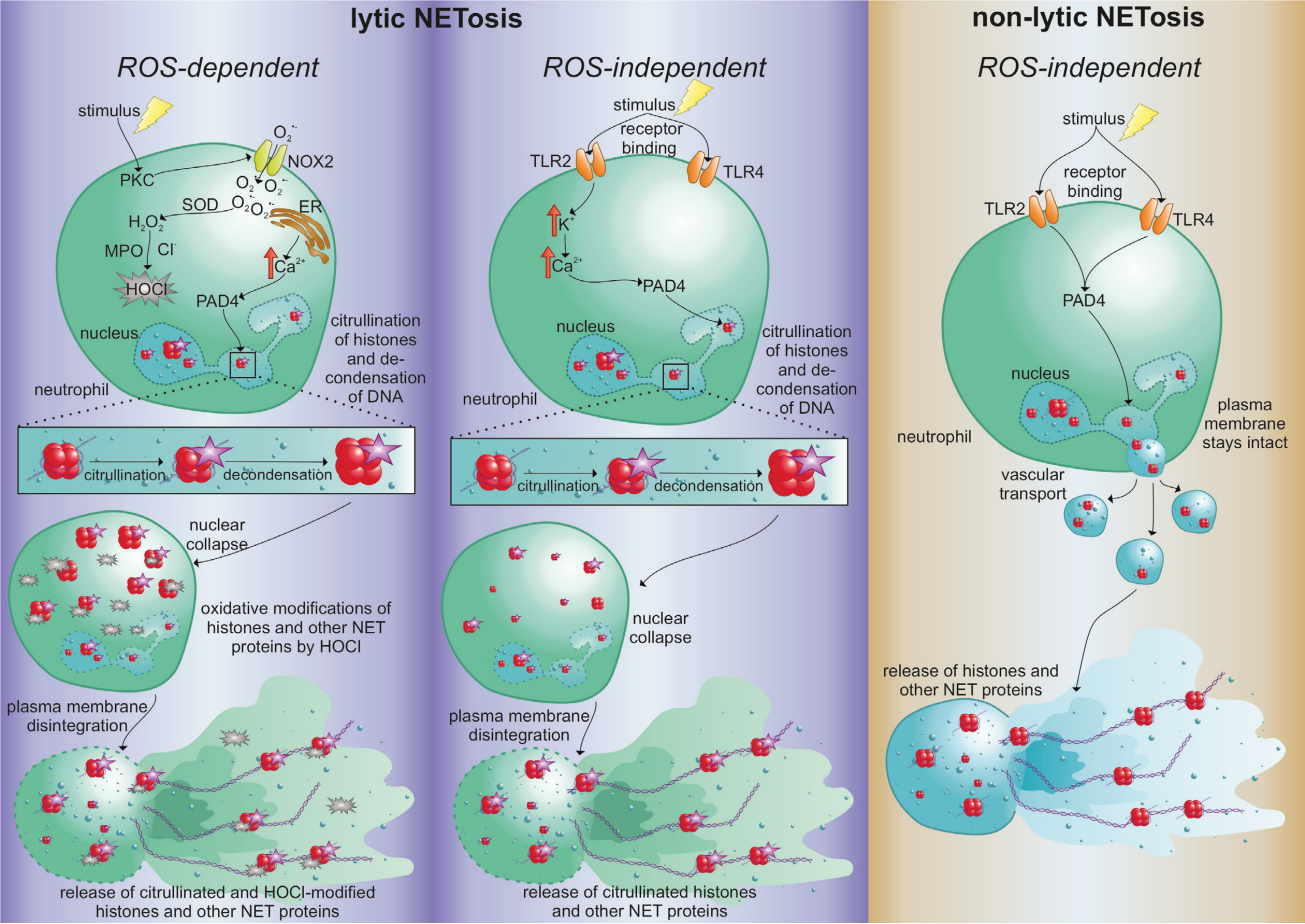
Histone release from immune cells via NETosis Histones are the most abundant proteins in extracellular traps and can be released by (1) reactive oxygen species (ROS)-dependent lytic, (2) ROS-independent lytic, and (3) ROS-independent non-lytic pathways. Each pathway typically involves activation of peptidyl arginine deiminase 4 (PAD4) triggered by elevated Ca^2+^, which citrullinates histones, promoting decondensation of the chromatin. Panel 1 shows activation of protein kinase C following stimulation, leading to an increase in Ca^2+^, the assembly of NADPH oxidase 2 (NOX2) and formation of superoxide (O_2_^−•^), which dismutates to hydrogen peroxide (H_2_O_2_) via the action of superoxide dismutase (SOD). Myeloperoxidase (MPO) produces hypochlorous acid (HOCl), which helps to break down the nuclear envelope, as well as contributing to chromatin decondensation. This results in oxidative modifications to histones as well as citrullination. Panel 2 shows the elevation of Ca^2+^ and activation of PAD4 via either receptor binding or the stimulus acting as an ionophore. Lytic NETosis follows disintegration of the plasma membrane and typically occurs over a period of 2–4 h. Panel 3 shows non-lytic NETosis, where the plasma membrane remains intact and only chromatin from the nucleus passes the plasma membrane into the extracellular space through the formation of intracellular vesicles. This pathway is very rapid, occurs independently of ROS formation, and is typically associated with infections [35–37]. It should be noted that other TLRs, pattern recognition receptors, complement receptors, Fc receptors, and chemokine receptors can also be involved in NETosis [38]. The figure was created with CorelDraw® 2019, version 21.2.0.708.

Histones can be released from the nucleus into the extracellular environment via different pathways, with cell death being responsible for most of these pathways, which include apoptosis [20,21], necrosis [22–25] pyroptosis [26] and ferroptosis [22,27] (Figure 1). There is also evidence for more specific, cell-type-dependent pathways, particularly from malignant, cancer cells, from exosome and amphisome formation (Figure 2) [28,29]. Interestingly, neutrophils can also release histones and DNA via exosome formation [30]. However, the release of extracellular traps (ETs) by neutrophils, macrophages, eosinophils and other immune cells is one of the most important mechanisms responsible for histone release, particularly in pathological conditions (Figure 3) [31–34].

### Apoptosis, pyroptosis, necrosis, necroptosis, and ferroptosis

Histones can enter the extracellular environment following apoptosis, which is associated with cell shrinkage and the formation of apoptotic bodies, resulting from activation of caspases, that are released in vesicles (Figure 1) [39,40]. However, in general, this is a targeted, non-inflammatory pathway, as the vesicles are taken up by phagocytes, which limits damage to the surrounding tissue [40]. This can be an important pathway to eliminate genotoxic, damaged cells, as displacement of histones (e.g. histone H1.2) from chromatin induces the mitochondrial release of cytochrome *c*, as the cell is alerted to the DNA damage [14,41].

In contrast, the release of histones through other cell death pathways like necrosis, necroptosis, pyroptosis, and ferroptosis, which are observed in multiple cell types, often occur under inflammatory conditions and are linked with promoting tissue damage [24,25]. Necrotic cells and their organelles swell due to disrupted ion pumps (oncosis), which culminates in membrane damage and rupture, causing histones and other cell contents to be released into the extracellular environment in a non-controlled manner (Figure 1) [39]. This pathway of release of cell contents usually triggers a further inflammatory reaction, which can then lead to damage to neighbouring cells and host tissue [24,25]. However, a secondary necrosis pathway could also be activated in apoptotic cells due to the caspase cleavage of gasdermin E (GSDME) and pore formation followed by membrane lysis [42]. This pathway remains controversial, as GSDME is not involved in regulating secondary necrosis in human T cells and monocytes or acting as a negative regulator of apoptotic cell disassembly in these models [43].

Microbes, together with molecules recognised as danger signals, can induce a programmed form of necrosis termed pyroptosis [44,45]. This release pathway of histones also involves pore formation in the plasma membrane, but in this case, it is driven by caspase-dependent cleavage of gasdermin D (GSDMD) activated by binding to toll-like receptors (TLRs) or NOD-like receptors (NLR) and non-canonical inflammasome formation (Figure 1). This promotes cell swelling, lysis, and the release of pro-inflammatory cytokines (interleukin (IL)-1β, IL-18) and simultaneous breakdown of DNA by endonucleases [44,45]. Different types of cells can undergo pyroptosis, including macrophages [46], neutrophils [47], epithelial and endothelial cells [48], and hepatocytes [49]. Not all cells are proven to release histones during this process, but importantly, extracellular histones can trigger this response in several cell types [46,49,50].

When immune and inflammation response mediators bind to death receptors (e.g. TNF receptor 1, TLR-3/4, or interferon receptor) and the apoptotic cell death pathways are compromised because of inhibition of caspases or apoptotic proteins, for example, cell death can be initiated via necroptosis [51,52]. A RIPK1–RIPK3–MLKL complex (necrosome) is formed due to inactivity of caspase and absence of apoptotic proteins. RIPK3 (receptor-interacting protein kinase 3) phosphorylates MLKL (mixed lineage kinase domain-like), which leads to a dissociation of phosphorylated MLKL from the necrosome and translocation to the plasma membrane, where MLKL mediates pore formation and plasma membrane damage resulting in the release of DAMPs, which can include histones [51,52].

Ferroptosis results in the release of histones by a regulated form of cell death involving iron, which promotes lipid radical formation and peroxidation on cell membranes (Figure 1) [53]. This occurs when an accumulation of iron or depletion of antioxidant defenses, particularly glutathione and glutathione peroxidase 4 allow lipid peroxides to accumulate and trigger ferroptosis through membrane instability and disturbed protein homeostasis [53]. Ferroptosis is reported in a range of cell types, including endothelial cells, fibroblasts, macrophages, dendritic cells, T-lymphocytes [54], neutrophils, pancreatic β-cells [55], neurones [56], cancer cells [57], cardiomyocytes [58], hepatocytes [59], and kidney tubular epithelial cells [60]. In general, there is a lack of direct evidence for the specific release of histones in this context, but ferroptosis can be induced by extracellular histones, for example, by histone H3 during liver failure [61].

### Immune cell release of extracellular traps (ETosis)

The release of extracellular traps by neutrophils (NETs) has been examined extensively since the initial discovery of this distinct type of cell death [62], which is characterised by release of decondensed chromatin containing histones and other proteins often with an antimicrobial function, as a means to trap and kill bacteria [63,64]. This pathway is not unique to neutrophils, but also occurs in other types of immune cell, including eosinophils (EETs) [34], macrophages (METs) [33] and monocytes [65] and mast cells (MCETs) [66]. A schematic overview of the key steps involved in lytic (suicidal) and non-lytic (vital) NETosis, which differ by both timing, occurring over a few hours or minutes, respectively, as well as the overall fate of the cell is shown in Figure 3 [32,67]. There are several different surface receptors involved in NETosis, particularly pattern recognition receptors, including TLRs, NRLs, and C-type lectin receptors, complement receptors, Fc receptors and chemokine receptors [38]. A detailed description of the mechanisms involved in stimulating ET release is beyond the scope of the current article, and the reader is referred to other specialist reviews (e.g. [32,67,68,69]).

In brief, it is well established histones are the most abundant proteins in NETs [8,70–73], EETs [68,74], and METs [75]. Citrullination of the histones is a key step in the release pathway, with both suicidal and vital NETosis, which is driven by the activation of PAD4 by elevated Ca^2+^ concentrations (Figure 3). It has been reported that NET release can also occur following activation of PAD4 by MLKL during neutrophil cell death by necroptosis [76] or apoptosis [77]. In the case of apoptosis, this primes the neutrophils for NET release following membrane disruption by GSDME [77]. Similarly, NET formation has been linked with GSDMD-mediated pyroptotic cell death in neutrophils [47,78]. It is proposed that the resulting citrullination of Arg residues on histones, particularly H3 and H4, promotes the decondensation of chromatin that precedes NETosis [32,79]. However, ATAC-see, which is a method combining *in situ* transposase-mediated fluorescent labelling with sequencing to image and map open chromatin, showed that NET release from neutrophils stimulated with 12-myristate 13-acetate (PMA) was divided into two distinct steps [80]. PAD4-mediated histone citrullination was required only for the second step, where mononucleosomes were disassembled into free DNA and histones before release as NETs [80]. This has led to the wide use of citrullinated histones as markers of NETs and the therapeutic targeting of PAD4 to limit extracellular histone release [14,19]. However, it should be noted that there are examples of PAD-independent NETosis, which are relevant to consider, including in a pathological context [81,82].

Other enzymes, including NOX2 and MPO, are also known to play a role in the release of NETs [67,83,84]. This has led to a further designation of ROS-dependent and -independent release mechanisms (Figure 3). The ROS-dependent pathway can be triggered by various pathogens but also by different inflammatory stimuli, particularly PMA and cytokines like tumour necrosis factor α (TNF-α) and IL-1β [85]. It is proposed that TNF-α and IL-1β bind to their respective transmembrane receptors (TNFR and IL-1R) on the neutrophil surface, which activates downstream protein kinase cascades that increase ROS production [86]. The elevation of ROS during NETosis, particularly the MPO-derived oxidant HOCl, plays a role in breaking down the nuclear envelope and facilitating the unfolding of chromatin [67,83,84] but can also promote the oxidative modification of histones on NETs (see also below) [8].

## Post-translational modifications of histones

Histones are susceptible to PTMs, and in the nucleus, these modifications are often considered epigenetic marks and have significant functional implications [87]. Some common histone PTMs occurring in the nucleus include methylation of Arg and Lys, acetylation and ubiquination of Lys, and phosphorylation of Ser, Thr, and Tyr [3,4,10,88]. These modifications take place mainly at the N-terminus of the histones and affect DNA binding, chromatin folding, and the accessibility of other DNA-binding proteins [4,10]. This influences various DNA template processes, such as replication or transcription of DNA, resulting in functional effects on gene expression or regression [89]. There is growing evidence for a role of oxidative modifications on nuclear histones, including 4-hydroxy-nonenal [90,91], and others resulting from exposure to ROS or reactive nitrogen species, which promote structural alterations, partial unfolding and thermal destabilisation [10,11]. Depending on the nature of the modification, this can lead to changes in histone transport into nucleosomes [92], and/or nucleosomal assembly [93], structure and stability [94], and ultimately altered gene expression patterns [10,11].

Other histone PTMs, particularly citrullination of Arg, are involved in the chromatin decondensation that precedes release of NETs (Figure 3), resulting in their detection in the extracellular environment. Some examples of histone citrullination relevant to different diseases are given in Table 1, together with studies showing histone carbamylation, which is also related to NET release [95,96]. In this case, carbamylation is attributed to the conversion of Lys to homocitrulline, which results presumably from reactions driven by cyanate [95,96], which may be linked to uremia or involve the action of MPO and thiocyanate [97]. In addition to citrullination and carbamylation, acetylation and methylation of histones have also been reported on NETs, which could be important in influencing their reactivity when present outside the cell [8,98,99].

**Table 1:** Overview of studies detecting histone citrullination and carbamylation *in vivo*

PTM	Location	Disease	Reference
CitH3	Plasma	Sepsis	[100]
CitH3	Serum	Septic shock	[101]
CitH3	Neutrophils isolated from plasma	COVID-19	[102]
CitH3	Carotid atherosclerotic plaques	Chronic brain ischemia or ischemic stroke	[103]
CitH3	Plasma, blood, tissue	Abdominal aortic aneurysms	[104]
CitH3, CarH4	Serum, gingival crevicular fluid	Periodontitis	[95]
CarH3, CarH4	Plasma, synovial fluid	Rheumatoid arthritis	[96]
CitH3	Serum	Dermatomyositis	[105]

CitH3: citrullination of H3; CarH3: carbamylation of H3; CarH4: carbamylation of H4.

Lastly, recent studies have provided evidence for the modification of histones on NETs by MPO-derived HOCl [8]. MPO binds to the acidic patch of the histone nucleosome, which has a destabilising effect [106], and may contribute to the close localisation of the histones and MPO on the NET backbone [107]. HOCl targets the abundant Lys residues of histones forming unstable *N*-chloramines, which decompose to nitriles or Lys carbonyl groups (aminoadipic semialdehydes) and chlorinates Tyr residues forming 3-chlorotyrosine [7,9]. The chlorination of Tyr-88 on histone H4 is a particularly abundant modification associated with NETs [8].

## Extracellular reactivity of histones

Extracellular histones, either alone or in combination with chromatin, play a central role in inflammation and immune reactions [108]. The recognition of extracellular histones as DAMPs by pattern recognition receptors, particularly TLR2, TLR4, and NLRs, is both beneficial, as recruitment of immune cells and amplification of inflammation helps to clear infection, but if sustained, can also be highly damaging to the host. Thus, the ability of extracellular histones to bind to, and disrupt the structure of, cell membranes has opposing effects. This is because as the cytotoxic, lytic properties of histones are not limited to pathogens, and there is also significant collateral damage to host tissues, which contributes to many diseases. Thus, through binding to cell surface receptors and/or by interacting directly with cell membranes and coagulation factors, extracellular histones can promote inflammation, cytotoxicity, and coagulation. Some examples of the consequences of these reactions are outlined below, with a particular focus on those relating to inflammatory pathologies, where the modification of histones is particularly relevant.

### Beneficial reactions

Histones have been known for their antimicrobial properties for more than 80 years [109]. The growth of Gram-positive and Gram-negative bacteria can be inhibited and their survival compromised by both linker (H1) and core histones (H2A, H2B, H3, and H4) destroying the cell wall by charge changes, penetrating the membrane, or histones binding to DNA, leading to inhibition of transcription [110–115]. A summary of the minimum inhibitory concentrations (MIC) of histones required to inhibit bacterial growth and kill various Gram-positive and Gram-negative bacteria is collected in Table 2. It is interesting to note that, depending on the histone preparation, origin and composition of the different histone types, the MIC, minimum bactericidal concentration (MBC), and half maximal inhibitory concentration (IC_50_) can vary slightly, which may reflect the abundance of the positively charged Lys and Arg residues [112–114]. Marsman *et al.* have demonstrated the clinical relevance of histones, particularly histone H1, in fighting infections by demonstrating the presence of this histone in abscess biopsies from methicillin-resistant *Staphylococcus* *aureus* (MRSA)-infected patients [115]. Under physiological conditions, H1 shows a significantly stronger effect in killing MRSA compared with core histones, perhaps reflecting its ability to bind more strongly to the bacterial membrane in this case [115]. The importance of histone-membrane interactions is further highlighted in studies by Li *et al.*, who demonstrated that free histones, rather than the core histones packed into nucleosomes, were able to kill bacteria more effectively [116].

**Table 2:** Histone concentrations needed to inhibit growth in different types of bacteria

Bacteria	Histone origin	Histone types	Minimum inhibitory concentration (MIC)	Minimum bactericidal concentration (MBC)	Half maximal inhibitory concentration (IC_50_)	Reference
**Gram-positive**						
***Bacillus subtilis***	Chicken erythrocytes	Mix of H1, H2A, H2B, H3, H4, H5	3 ± 1 μg/ml	-	-	[113]
***Enterococcus faecalis***	Chicken erythrocytes	Mix of H1, H2A, H2B, H3, H4, H5	700 ± 100 μg/ml	1100 ± 200 μg/ml	-	[113]
***Staphylococcus aureus***	Chicken erythrocytes	Mix of H1, H2A, H2B, H3, H4, H5	6 ± 1 μg/ml	-	-	[113]
**Biofilm methilicin-resistant (MRSA)**	Chicken erythrocytes	Mix of H1, H2A, H2B, H3, H4, H5	8 ± 2 μg/ml	8 ± 3 μg/ml	-	[113]
	Chicken erythrocytes	H1 (9.7% ± 1) H2A (15% ± 1) H2B (19% ± 1) H3 (24% ± 1) H4 (14.6% ± 0.4) H5 (18% ± 1)	8 ± 2 μg/ml	-	-	[114]
	Chicken erythrocytes	H5 (96.8%)	2.4 ± 0.8 μg/ml	2.6 ± 1.1 μg/ml	-	[114]
**Biofilm methicilin-sensitive *S. aureus* (MSSA)**	Chicken erythrocytes	Mix of H1, H2A, H2B, H3, H4, H5	6 ± 1 μg/ml	6 ± 1 μg/ml	-	[113]
	Chicken erythrocytes	H5 (96.8%)	3.8 ± 0.4 μg/ml	4.0 ± 0.0 μg/ml	-	[114]
**Gram-negative**						
***Salmonella typhimurium***	Chicken erythrocytes	Mix of H1, H2A, H2B, H3, H4, H5	3.6 ± 0.4 μg/ml	-	-	[113]
***P*** ***seudomonas aeruginosa***	Chicken erythrocytes	Mix of H1, H2A, H2B, H3, H4, H5	5 ± 1 μg/ml	-	-	[113]
***Escherichia coli***	Chicken erythrocytes	Mix of H1, H2A, H2B, H3, H4, H5	21 ± 3 μg/ml	-	-	[113]
	Calf thymus histones	H2B	-	-	3.8 μM	[112]
	Calf thymus histones	H3	-	-	10 μM	[112]
	Calf thymus histones	H4	-	-	12.7 μM	[112]

The inhibition of bacterial growth is dependent on histone type or composition of histone mixture and bacteria type.

In addition to the antimicrobial properties of histones, recent studies have highlighted a copper reductase role for the histone H3–H4 tetramer, which is postulated to be important in maintaining copper homeostasis [117]. Thus, the His-113 and Cys-110 residues of histone H3, when present in H3–H4 tetramers, are proposed to form a metal-binding site that facilitates the reduction of Cu^2+^ to Cu^+^ [117]. The reductase function is lost on mutation of these residues, which is linked to defective mitochondrial respiration and decreased activity of SOD1 [117]. This copper reductase activity of H3–H4 tetramers is also observed with intact nucleosomes, where there is dependence on Zn^2+^ for activity [118]. It is not known whether these reactions of histones have relevance in the extracellular environment, as the conformation of the H3–H4 tetramer of the nucleosome is important for forming the putative Cu^2+^ binding site. This ability of histones to perturb the redox state of copper could be detrimental in the extracellular environment by promoting tissue damage via Fenton-like chemistry and ROS production. Similarly, the ability of extracellular histones to induce eryptosis [119] and erythrocyte fragility [120] could further promote damaging Fenton reactions via increasing the release of iron [121].

### Detrimental and damaging reactions

The reactivity of extracellular histones with endothelial cells has been studied widely, owing to the relevance of these reactions, particularly in acute inflammatory pathologies like sepsis [122–125]. Histones can directly bind to membrane phospholipids, triggering the influx of Ca^2+^ into the cell, leading to dilation, endothelial dysfunction, and ultimately causing cell death [15,126]. Recent studies show that the globular domains of core histones (H2A, H2B, H3, and H4) drive pore formation in cell membranes, resulting in lytic cell death, whereas the N-terminal histone tails alone are not cytotoxic [15]. Histone-induced lytic cell death is also important in the context of atherosclerosis, where it has been demonstrated that extracellular histone H4 from NETs binds to, and lyses, smooth muscle cells (SMCs) [127]. This promotes plaque destabilisation, which could be decreased by activated protein C proteases that neutralise histone H4 [127]. Extracellular histones can increase calcification of SMCs by promoting a phenotype shift to favour the increased expression of osteoblast marker genes [128], which could also be important in lesion development in atherosclerosis.

In addition to promoting lysis, extracellular histones can amplify inflammation and dysfunction in these vascular cell types by activating different signalling cascades, which can involve the interaction with cell surface receptors, particularly TLR2 and TLR4. There is evidence for the activation of the NLRP3 inflammasome and apoptotic or pyroptotic cell death in SMCs [129] and endothelial cells [124,130]. In SMCs, up-regulation of Forkhead box protein O4, as a result of activation of 5′-AMP-activated protein kinase (AMPK) signalling is important [129]. Activation of AMPK has also been implicated in endothelial cells exposed to histones, which, together with AKT, is linked to the induction of autophagy and apoptosis [131]. Exposure to histones can also decrease the activity of endothelial nitric oxide synthase and nitric oxide production [132] and increase the expression of tissue factor [133]. The up-regulation in the expression of tissue factor is attributed to activation of NF-κB and AP-1 from histone binding to TLRs [133]. These reactions are also important *in vivo*, where histone binding to TLR2 and TLR4 activates endothelial cells and triggers pro-inflammatory reactions in mouse models of acute renal failure and acute pulmonary failure [134,135]. Here, the interaction with TLR4 was only observed with Arg-rich histones H3 and H4, rather than the Lys-rich core histones H2A and H2B [135].

In addition to activating endothelial cells, there is evidence that extracellular histones can promote the activation of immune cells to further elevate inflammation and potentially tissue damage [136]. Histone H4 alters the membrane permeabilisation of neutrophils, which causes a sustained increase in intracellular Ca^2+^ that culminates in respiratory burst activation and degranulation [137]. In macrophages, the histone-induced influx of Ca^2+^ is shown to promote externalisation of the TWIK2 K^+^ channel on the plasma membrane, leading to activation of the NLRP3 inflammasome [138]. Histone binding to TLRs is also important in driving immune cell dysfunction. Thus, histones released by NETs amplify the differentiation of T-helper-17 (Th17) cells by binding to TLR2 and activating MyD88 (myeloid differentiation primary response 88) [139]. These observations were replicated in experiments with recombinant histones, where it was further demonstrated that Th17 cell differentiation occurs through RORγt (RAR-related orphan receptor gamma) as a consequence of activation of STAT3 downstream of TLR2 [139]. These effects with recombinant histones were inhibited by β-methyl-cellobioside sulfate, which lacked efficacy towards other TLR2 agonists [139]. In addition, interactions with TLR4 are important in driving inflammatory signalling and cytokine release in monocytes [140]. Histones can also be neurotoxic, with evidence for damage to cortical neurons [141], microglia [142], and a range of other nerve cell types [143], and amplify inflammatory responses and cell death in adipocytes [144].

Lastly, extracellular histones can interact with coagulation factors such as prothrombin, FXa and FSAP (FVII activating protease), which leads to the activation of platelets and the formation of fibrin and thrombin, thereby inducing coagulation, which can result in thrombocytopenia and hypercoagulation [145–147]. This is exacerbated by the ability of histones to up-regulate the endothelial expression of tissue factor [133] and directly activate platelets [147]. Thus, it has been demonstrated in mice that a sub-lethal histone infusion results in rapid depletion of platelets [145]. These reactions are also believed to be important in humans, on the basis of *ex vivo* studies with commercial histones [148], and activation of coagulation factors, such as FSAP at pathologically relevant concentrations of histones *in vitro* [149]. Similarly, there is an inverse correlation between extracellular histone H3 in the circulation with antithrombin levels and platelet counts, which is linked to mortality in patients with sepsis [150]. This has led to significant interest in developing therapeutic approaches to limit histone-induced coagulation [14,146].

## Influence of histone PTMs in driving cellular damage

The effect of histone PTMs on the extracellular reactivity of histones has not been studied extensively, in contrast with their known role in altering DNA binding ability in the nucleus. However, modifications of histones can affect both charge and structure, which will influence their reactivity when present outside the cell, similarly to interactions in the nucleus. This has importance considering the role of citrullination in the release of NETs, for example, which are an important source of extracellular histones, particularly in inflammatory pathologies [32]. While citrullination of histones has been widely documented *in vivo* (Table 1), relatively few studies have considered the implications of this (and other types of) modification on histone extracellular reactivity. Distinguishing whether different histone PTMs influence cellular reactivity also has implications for the development of more specific histone-targeted therapies.

Interestingly, *in vitro* studies show that citrullination of histones can both decrease their cytotoxicity and perturb inflammatory signaling [140,100]. In monocytes, citrullination decreased the histone cytotoxicity but potentiated binding to TLR4 and activation of inflammatory signalling and cytokine release [140]. This study also reported a novel synergistic relationship between histones, which bind and activate TLR4, and DNA, which facilitates the recruitment of TLR4, at sub-lethal histone concentrations [140]. The ability of citrullinated histones to increase the in pro-inflammatory immune response was also demonstrated *in vivo*, by alterations in plaque formation in atherosclerosis-prone mice [140]. Citrullinated histones are also less cytotoxic to endothelial cells compared with histones lacking this modification [100]. Again, citrullinated histones could induce an inflammatory response in the endothelial cells, shown by cytokine release and the expression of cellular adhesion molecules [100]. Other studies have shown that citrullination of histone H3 (CitH3) causes microvascular leakage and disruption of the endothelial cell barrier by opening adherens junctions and reorganising the actin cytoskeleton [151]. In this case, there was no direct comparison with native histone H3, so it is not certain whether citrullination potentiates the endothelial cell barrier disruption. However, it is notable that this effect of CitH3 was observed in the absence of cell death [151], which would be expected on exposure of endothelial cells to a comparable concentration of non-modified histone H3.

Similar effects were observed with acetylation [124] and HOCl-induced modification of extracellular histones [152,153]. Like citrullination, these histone modifications are important to consider in inflammatory pathologies, as they are also associated with NETs [8,98,99]. Hyperacetylation of histones when present in the extracellular environment showed an apparent protective effect on cell viability and decreased the extent of cell death seen with endothelial cells compared with native histones [124]. Interestingly, in this case, the expression of some antioxidant and NLRP3 inflammasome-related genes was down-regulated compared with that seen with non-modified histones [124]. As such, alteration of the balance of histone acetylation and deacetylation by use of histone deacetylase inhibitor (HDACi) drugs could be an alternative therapeutic approach to modulate acetylated histone reactions in the extracellular environment. However, given the crucial role of histone deacetylases in regulating gene expression and influencing chromatin remodeling and the potential for off-target effects, this approach is likely to be challenging.

The modification of histones by the MPO-derived oxidant HOCl also decreased the extent of histone cytotoxicity in studies with SMCs [153] and INS-1E cells as a β-cell model [152]. In each case, the decrease in histone toxicity was related to the concentration of HOCl and hence the extent of histone modification. With both cell types, HOCl-modification of the histones had either no effect or a slight stimulatory effect on inflammatory and stress-related signalling compared with the non-modified histones [152,153]. Extracellular histones also decreased the expression of INS-1 and INS-2 genes and insulin content in the INS-1E cells, which was more pronounced on pre-treatment of the histones with HOCl [152]. This may have relevance in the development of type 2 diabetes, where NETs are elevated in the circulation [154,155]. It is not known which histones are responsible for the alterations in cellular function in the studies with HOCl-modified proteins, as a commercial mixture of histones containing core and linker histones was used, which is a limitation [152,153]. The reaction with HOCl results in the different oxidative PTMs on histones, including oxidation of Met, Lys-derived nitriles and carbonyls (aminoadipic semialdehydes) [7], and chlorination of Tyr [7,9], which are also observed on histones in NETs [8]. These modifications, like citrullination and acetylation, will affect the charge and structure of the histones.

The mechanisms responsible for the decreased cytotoxicity and differential effects on pro-inflammatory capacity seen in cellular studies with different types of modified histones are not well understood. It has been proposed that in each case, the presence of PTMs alters the charge of the histone, particularly on modification of Lys or Arg residues, which influences the interactions with cell membrane and surface receptors [124,140,152,153]. While in general, histone PTMs appear to be associated with a lower cytotoxicity compared with native histones, the extracellular reactivity is also influenced by both the nature and extent of modification and the specific cell type under study. In addition, the results from recent proteomic studies show that histones H3 and H4 on NETs contain a combination of PTMs, with evidence for acetylation of Lys, oxidation of Met, citrullination of Arg, and chlorination of Tyr [8]. It is likely that the presence of a combination of histone PTMs might have synergistic or even opposing effects, which has not been studied. More work is needed to understand these interactions in more detail and to assess how the presence of DNA affects reactivity, which is relevant to NET-related histones.

## Conclusions

There is compelling evidence for a key role of extracellular histones, particularly when released from neutrophils as NETs, in driving the development of a wide range of acute and chronic pathologies [12,13]. Extracellular histones promote cytotoxicity, inflammation and coagulation, which can culminate in organ failure, as highlighted by the association of the levels of circulating histones with poor prognosis and mortality in patients with sepsis [100,156,157]. This has led to a significant interest in the development of therapeutic approaches to target histones [14,19]. However, while NETs are an important source of extracellular histones, the effect of citrullination and other NET histone PTMs on their reactivity outside the cell has not been widely studied. Recent data suggest that the modification of histones outside the cell may not necessarily be protective, despite decreased cytotoxicity compared with native histones, because increased cell survival may facilitate cell damage by other pathways, including elevated inflammatory signalling [140,100,152,153] or altered endothelial barrier function [151]. Thus, there may be therapeutic value in the development of interventions to specifically target modified histones, highlighted by studies showing that the outcomes of systemic and chronic inflammation can be improved by treatment with CitH3 targeted antibodies [158] and PAD inhibitors [140,159].

## Perspectives

- There is increasing evidence that modification of histones influences their reactivity in the extracellular environment, which could play a role in a range of acute and chronic pathologies.
- Gaining a greater understanding of the cellular mechanisms involved in the differential reactivity of modified histones could be useful therapeutically and improve our understanding of inflammatory processes in health and disease.
- More work is needed to examine how specific histone modifications, and their combination, influence their interactions with membranes and surface receptors on host cells and different pathogens.

## Open Access

Open access for this article was enabled by the participation of the University of Copenhagen in an all-inclusive Read & Publish agreement with Portland Press and the Biochemical Society under an agreement with the Royal Danish Library.

## Abbreviations

AMPK: 5′-AMP-activated protein kinase
CarH3: carbamylation of H3
CarH4: carbamylation of H4
CitH3: citrullination of H3
DAMPs: damage-associated molecular patterns
EET: eosinophil extracellular trap
ET: extracellular trap
FASP: FVII activating protease
GSDMD: gasdermin D
GSDME: gasdermin E
HOCl: hypochlorous acid
IL: interleukin
MBC: minimal bactericidal concentration
MCET: mast cell extracellular trap
MET: macrophage extracellular trap
MIC: minimum inhibitory concentration
MLKL: mixed lineage kinase domain-like
MPO: myeloperoxidase
MRSA: methicillin-resistant *S. aureus*
NET: neutrophil extracellular trap
NLR: NOD-like receptor
NOX2: NADPH oxidase 2
PAD4: peptidyl arginine deiminase 4
PMA: 12-myristate 13-acetate
PTM: post-translational modification
RIPK3: receptor-interacting protein kinase 3
ROS: reactive oxygen species
SMC: smooth muscle cell
SOD: superoxide dismutase
Th17: T-helper-17
TLR: toll-like receptor
TNFα: tumour necrosis factor alpha
